# Functional lung MRI for regional monitoring of patients with cystic fibrosis

**DOI:** 10.1371/journal.pone.0187483

**Published:** 2017-12-07

**Authors:** Till F. Kaireit, Sajoscha A. Sorrentino, Julius Renne, Christian Schoenfeld, Andreas Voskrebenzev, Marcel Gutberlet, Angela Schulz, Peter M. Jakob, Gesine Hansen, Frank Wacker, Tobias Welte, Burkhard Tümmler, Jens Vogel-Claussen

**Affiliations:** 1 Department of Diagnostic and Interventional Radiology, Hannover Medical School, Hannover, Germany; 2 Biomedical Research in Endstage and Obstructive Lung Disease (BREATH), German Center for Lung Research, Hannover, Germany; 3 Department of Pediatric Pulmonology, Allergology and Neonatology, Hannover Medical School, Hannover, Germany; 4 Department of Experimental Physics 5, University of Würzburg, Würzburg, Germany; 5 Department of Respiratory Medicine, Hannover Medical School, Hannover, Germany; University Children's Hospital Bern, SWITZERLAND

## Abstract

**Purpose:**

To test quantitative functional lung MRI techniques in young adults with cystic fibrosis (CF) compared to healthy volunteers and to monitor immediate treatment effects of a single inhalation of hypertonic saline in comparison to clinical routine pulmonary function tests.

**Materials and methods:**

Sixteen clinically stable CF patients and 12 healthy volunteers prospectively underwent two functional lung MRI scans and pulmonary function tests before and 2h after a single treatment of inhaled hypertonic saline or without any treatment. MRI-derived oxygen enhanced T_1_ relaxation measurements, fractional ventilation, first-pass perfusion parameters and a morpho-functional CF-MRI score were acquired.

**Results:**

Compared to healthy controls functional lung MRI detected and quantified significantly increased ventilation heterogeneity in CF patients. Regional functional lung MRI measures of ventilation and perfusion as well as the CF-MRI score and pulmonary function tests could not detect a significant treatment effect two hours after a single treatment with hypertonic saline in young adults with CF (p>0.05).

**Conclusion:**

This study shows the feasibility of functional lung MRI as a non-invasive, radiation-free tool for monitoring patients with CF.

## Introduction

Cystic fibrosis (CF) is an autosomal recessive, monogenetic multi-organ disorder. Airway disease determines morbidity and prognosis in most patients [1]. CF-causing mutations in the CF transmembrane conductance regulator (CFTR) lead to impaired chloride and bicarbonate secretion [2] which results in the dehydration of the airway surface liquid layer, mucus plugging, and airway obstruction [3].

Therapeutic interventions to improve mucus clearance are a cornerstone of treatment in CF [4]. Such interventions include regular chest physiotherapy, mucolytics, and also aerosolized hypertonic saline (HTS; 3% to 7% NaCl) [5]. Inhalation of HTS has been shown in clinical trials to significantly improve mucociliary clearance and ventilation homogeneity [6–9]. However, the immediate treatment effects of a single inhalation with hypertonic saline remain unknown. An adequate technology to address this issue could be functional lung magnetic resonance imaging (MRI), which has recently been shown to be feasible and sensitive to regional lung function changes in CF patients [10–12]. In recent years efforts have been made to assess regional pulmonary function using lung MRI: Oxygen enhanced functional lung MRI exploits changes of the T1 times under normoxic and hyperoxic conditions related to a combination of ventilation, perfusion and diffusion capacity of the lung[13]. Ventilation-weighted Fourier decomposition lung MRI is a contrast agent free proton MR-based technique, which can depict regional lung ventilation, which has emerged in the last few years[14–17]. An established technique for assessment of pulmonary parenchymal perfusion is dynamic contrast enhanced (DCE) MRI. A 4D dataset of T1 weighed images with a high temporal resolution is acquired after injection of i.v. contrast medium, allowing calculation of different hemodynamic parameters including pulmonary parenchymal blood flow[18–20].

Therefore the aim of this study was to evaluate if functional lung MRI can detect and quantify regional parenchymal differences between adolescents with CF and healthy volunteers. Furthermore it was evaluated if quantitative functional lung MRI parameters can detect changes in regional lung function two hours after a single hypertonic saline treatment in comparison to spirometry and multiple breath washout (MBW).

Compared to healthy controls functional lung MRI detected and quantified significantly increased ventilation and perfusion heterogeneity in CF patients, however regional functional lung MRI measures of ventilation and perfusion as well as pulmonary function tests could not detect a significant treatment effect two hours after a single treatment with hypertonic saline in young adults with CF.

## Materials and methods

### Patient characteristics and study protocol

Sixteen patients with confirmed cystic fibrosis, age between 11 and 20 years, and twelve healthy volunteers were included into this prospective single-center case-control study. These patients were recruited during their outpatient visits in our outpatient clinic for cystic fibrosis. The Participant recruitment started in February 2013 and the last MR examination was obtained in December 2014.

Inclusion criteria were a confirmed diagnosis of cystic fibrosis in a clinically stable condition, a forced expiratory volume in one second (FEV1) of at least 40 percent predicted for age and gender, and an age between 11 and 20 years. Exclusion criteria were contraindications to MRI or contrast media, pregnant or breast-feeding women, cigarette smokers or patients with hypertonic saline treatment (HST) within the last 7 days. The local ethics committee (Institutional review board at Hannover Medical School) approved the study protocol. All participants or their legal guardians provided written informed consent. As this was initially thought to be a truly observational case control study for measuring regional lung function after a clinically indicated and well established treatment procedure with hypertonic saline in CF patients this study was not registered before enrolment of participants started. After review by the editorial board of PLOS ONE it was judged necessary to register this study (ISRCTN 11682934).

**Fig 1:**
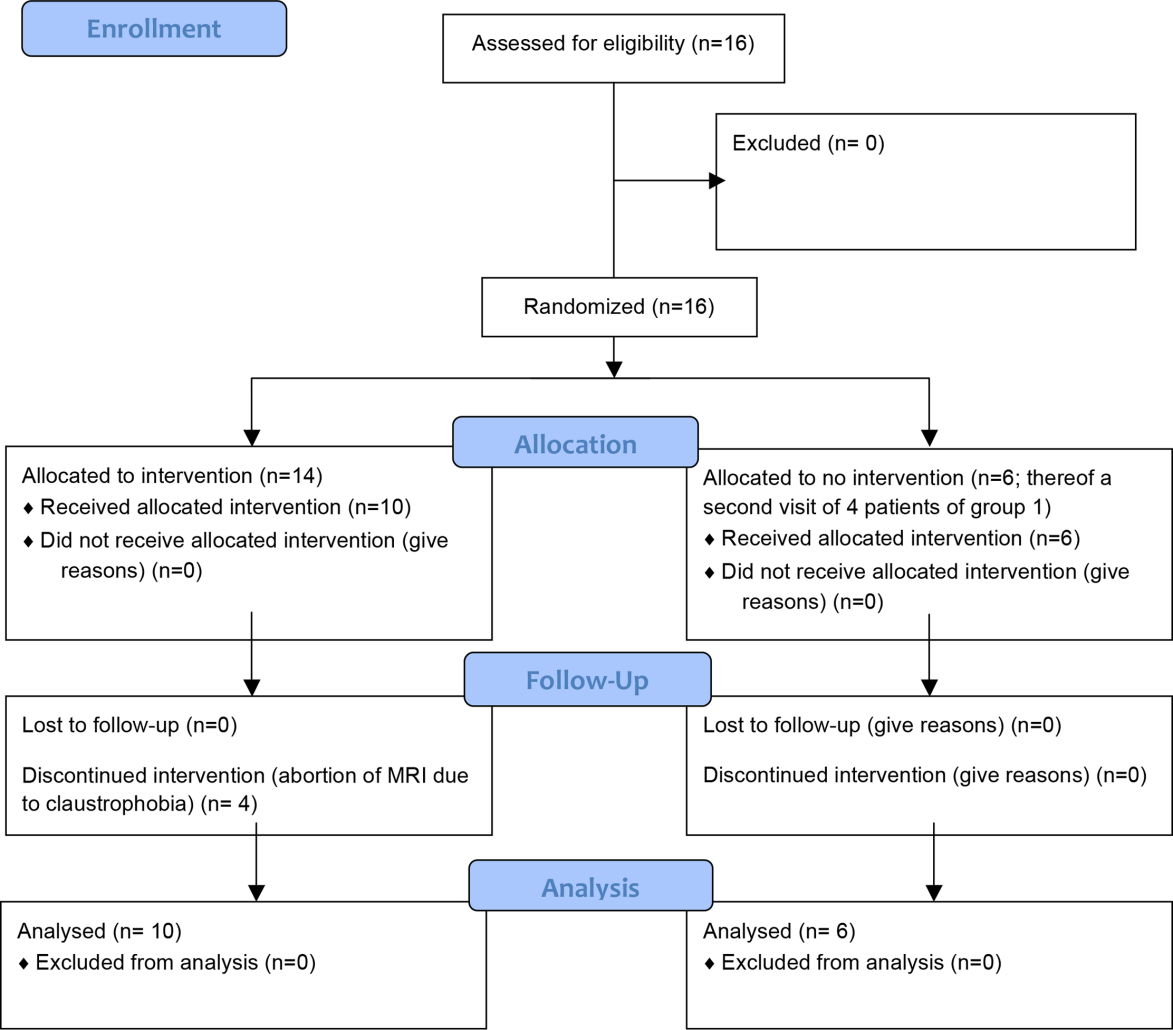
Patient cohort (CONSORT flow chart). Please note: For the control group a second visit of 4 patients of the treatment group were included resulting in 20 visits of 16 patients.

The CF cohort was divided into two groups: the treatment group (n = 14) received an inhalation treatment with inhaled hypertonic saline (7%), which was performed according to SOP 530.00 of the CFFT TDN (Therapeutic Diagnostic Network of the Cystic fibrosis Foundation) to produce induced sputum. The Inhalation treatment was carried out in the CF clinic by trained staff members. Neither patients nor the investigators were blinded regarding the inhalation treatment. Bronchodilators were not administered to exclude possible confounders. The control group (n = 6; a second visit of 4 patients of the treatment group were included) received no treatment (see Fig 1). MRI lung imaging at 1.5T (Avanto, Siemens Healthcare Erlangen, Germany) using an 8-channel torso phased array coil, multiple breath nitrogen washout and spirometry were performed before and two hours after a single treatment with inhaled hypertonic saline. An increased mucus clearance within 60 minutes and after 24 hours after HTS therapy was reported before [21]. Thus, in order to evaluate short-term improvements in lung function the second MRI examination was carried out two hours after HTS therapy.

**Fig 2:**
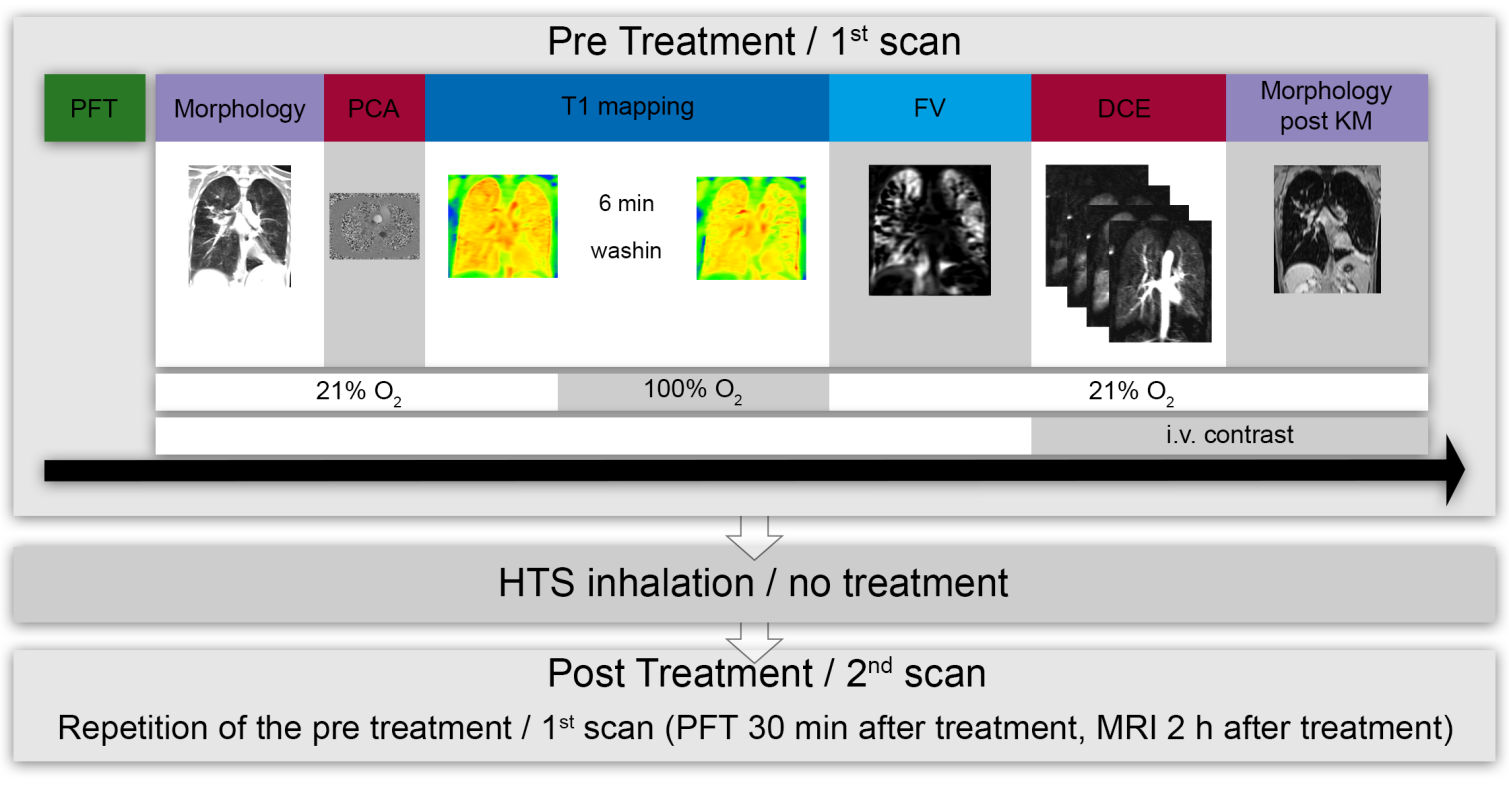
Study protocol. Pulmonary function testing (spirometry and multiple breath nitrogen washout (MBW)) were performed 60 to 90 minutes prior to the pre treatment scan / 1^st^ scan. MRI was performed as follows: first, morphological images were assessed followed by phase contrast angiography (PCA) in the ascending aorta. Afterwards T1 mapping breathing room air and again after six minutes of 100% oxygen wash-in time was acquired. Regional Fractional Lung Ventilation MRI (FV) was then acquired under normoxic conditions. Then for assessment of pulmonary parenchymal perfusion, dynamic contrast enhanced (DCE) MRI was carried out followed by a morphological sequence post i.v. contrast. Afterwards inhalation treatment with hypertonic saline (HTS, treatment group) or no treatment (control group) was performed and the PFT (30 minutes after treatment) and MRI (2 h after treatment) were repeated. Healthy volunteers underwent one scan using the same functional lung MRI protocol, except for DCE-MRI and phase-contrast MRI.

In addition, twelve healthy volunteers were included as controls, who underwent the same functional lung MRI protocol, except for dynamic contrast enhanced (DCE)-MRI and phase-contrast MRI. Fig 2 illustrates the study protocol.

Patient’s data was stored in the hospital information system, MR images were stored in the local picture archiving and communication system (PACS). Processed data was stored on a dedicated research server.

### Pulmonary parenchymal perfusion

Regional lung perfusion was evaluated using a three-dimensional DCE time-resolved angiography with stochastic trajectories (TWIST) MRI sequence in a single breath hold at end-inspiration as previously described [22]. MR imaging parameters were as follows: TE 0.7 ms, TR 2.1 ms, flip angle 25°, 40 three dimensional datasets with an update rate of 0.8–1.0 s, acquisition matrix 192 x 113, field of view 42 x 50 cm, 0.03 mmol/kg Gadoteric acid i.v.at 5 cc/s, 26–32 reconstructed coronal slices (slice thickness 6 mm) covering the whole lung. Pulmonary blood flow (PBF) maps were calculated by using a pixel-by-pixel deconvolution analysis and dedicated software (PMI-MIKE 0.4, Platform for Research in Medical Imaging [20]). The lobes of both lungs were manually segmented excluding the great central vessels. The middle lobe and lingula were combined with the corresponding upper lobes. Median parenchymal PBF and the quartile coefficient of dispersion (QCD) were calculated for the whole lung as well as each lobe separately. Phase contrast angiography (PCA) in the ascending aorta was carried out to assess cardiac output. This measurement was used to adjust PBF.

### Oxygen enhanced functional lung MRI

For rapid regional lung T1 mapping, an inversion-recovery snapshot fast low-angle shot sequence was used, which has been described in detail by Jakob et al [13], with parameters as follows: TE 0.8 ms, TR 3.0, flip angle 8°, 32 inversion times with a time frame of 200–6400 ms, acquisition matrix 128 x 64, field of view 50 x 50 cm. Three coronal slices were acquired with a slice thickness of 15 mm and a gap of 7.5 mm; the mid-slice was centered on the trachea. T1 maps were obtained in a single breathhold each at end-inspiration while the patient was breathing room air and again after six minutes of 100% oxygen wash-in time. One hundred% oxygen was administered at a flow rate of 15 L/min using a full closed face mask (Air Cushion Face Mask Size 3 or 4, VBM Medizintechnik, Sulz, Germany) with a filter (MicroGard II, CareFusion, Hoechberg, Germany) and a 2L reservoir (SPUR II, Ambu A/S, Ballerup, Denmark)[23]. T1 maps were calculated as previously described [13,23,24]: Registration of the individual magnitude images obtained at 100% oxygen onto room air images was performed by using a nonrigid registration algorithm (Advanced Normalization Tools (ANTS); *http://stnava.github.io/ANTs/* [25]). Afterwards T1 maps were calculated from the magnitude images by using a nonlinear fit [24] within a self-developed Matlab script [23](MATLAB 2012a, MathWorks, Natick, Mass). After manual segmentation, excluding the great central vessels, median T1 at room air, at 100% oxygen, Delta T1 and the QCD of each parameter were calculated for the whole lung and each lobe.

### Regional fractional lung ventilation MRI

Ventilation-weighted Fourier decomposition lung MRI was evaluated as previously described [15]. Three coronal slices positioned similar to T1 mapping were acquired using a spoiled gradient echo sequence with TE 0.7 ms, TR 3 ms, flip angle 8°, matrix size 128 x 96, field of view 50 x 50 cm, slice thickness 15 mm. Over a period of one minute at a temporal resolution of 288 ms, 200 images per slice were obtained. After a non-rigid image registration (ANTS) of the dynamic series of images to a reference image in mid position between end-inspiration and end-expiration a low-pass filter was applied to generate a series of ventilation-weighted images. Fractional ventilation (FV) was calculated by averaging the signals of the end-inspiratory (S_exp_) and end-expiratory images (S_Insp_) and using the following formula: FV = (S_Exp_ − S_Insp_)/S_Exp_ [26]. Lungs were segmented manually excluding the great central vessels. Median values and QCD were calculated for the whole lung and each lobe.

### CF-MRI-Score

As a visual semi quantitative assessment of CF associated lung changes a scoring system developed by Eichinger et al. was chosen [10]. Morphological sequences used for this readout were a balanced steady-state free-precession sequence and T2-weighted sequence with single-shot half-Fourier turbo-spin echo acquisition in the coronal and axial plane as well as a volume interpolated gradient echo sequence before and after i.v. contrast in the transversal plane [10,11]. A radiology resident with 2 years of experience in lung MRI (TFK) scored each examination twice within 2 months time between the 1^st^ and the 2^nd^ read, blinded to the previous results and pulmonary function test results.

### Pulmonary function tests

Spirometry and multiple breath nitrogen washout (MBW) [27] were performed 60 to 90 minutes prior to the first scan, and 30 minutes after treatment (i.e. 90 minutes prior to the second scan). Spirometry was performed with the Power-Cube Body+ instrument (Ganshorn Deutschland GmbH, Neuenkirchen, Germany). MBW tests were performed by ultrasonic technology using the EasyOne Pro LAB™ (ndd medical technologies AG, Zurich, Switzerland) following the protocol described by Fuchs et al.[28].

### Statistical analysis

The distribution of functional MRI parameters was tested for normality using the Shapirow-Wilk test. Since many of the tested variables were not normally distributed, nonparametric tests were used. Data are presented as median with 25^th^ and 75^th^ percentiles. A p-value of less than 0.05 was considered indicative of a significant difference.

Measurements in CF-patients and normal volunteers were compared using the Wilcoxon rank sum test. Receiver operating characteristic curve analysis was performed to evaluate the test performance of functional Lung MRI for discrimination between these two groups. For comparison of values pre- and post treatment, a paired two-sided Wilcoxon rank sum test was performed. Intraclass correlation coefficient (ICC) was calculated using the two-way random single measure model [29]. Spearman rho correlation was used. For statistical analysis JMP Pro 11 software (SAS Institute, North Carolina, U.S.A.) was used.

## Results

### Study participants

Sixteen patients (n = 10 treatment group; n = 6 control group) and twelve healthy volunteers completed a MRI examination without side effects (see Fig 1). Four CF patients (20%) did not complete the examination due to claustrophobia. A detailed list of patient’s demographics for the study groups is given in Table 1. The datasets of the pre-treatment scan of all CF patients of the treatment group (n = 10) and first scan of the CF patients of the control group, who had not participated in the treatment group (n = 2) were used for the comparison of CF patients with healthy volunteers.

**Table 1:** Subject demographics.

Characteristic		CF treatment	CF control	Healthy volunteers
No. of patients		14	6	12
No. of excluded participants		4	0	0
Reason for exclusion		Examination not completed		
Sex				
	No. of male subjects	2	1	7
	No. of female subjects	8	5	5
Age (y)		14.5 (12.75–16.75)	14.5 (13.25–16.25)	25.5 (22.25–35.25)
Body mass index (kg/m^2^)		18.2 (16.7–20.0)	17.8 (16.0–18.1)	21.3 (20.0–26.0)
FEV1 (% predicted)		85.0 (63.5–96.5)	79.0 (58.8–91.3)	
LCI		11.5 (10.4–14.3)	11.3 (7.8–16.8)	

Unless otherwise indicated data are median with 25th and 75th percentiles in parenthesis; FEV1—forced expiratory volume in 1 second, LCI–lung clearance index.

### Functional lung MRI is able to discriminate between CF-patients and healthy volunteers

Results of the MRI-scans of the CF cohort compared to healthy volunteers are presented in Table 2:

**Table 2:** Comparison of CF patients to healthy volunteers.

Variable		CF (n = 12)	Healthy volunteers (n = 12)	*P* value
Cardiac output (l/min)		5.8 (5.2–6.5)	-	
**Oxygen enhanced functional Lung MRI**				
Median T1 room air (ms)				
	whole lung	1186 (1104–1215)	1253 (1211–1281)	**0.002**
	upper lobes	1151 (1104–1151)	1257 (1213–1282)	**0.001**
	lower lobes	1207 (1112–1238)	1247 (1214–1283)	**0.019**
	ratio UL/LL	0.98 (0.92–1.01)	1.00 (0.99–1.02)	0.061
Median T1 oxygen (ms)				
	whole lung	1030 (1010–1077)	1099 (1057–1119)	**0.003**
	upper lobes	1025 (1000–1070)	1098 (1060–1126)	**0.002**
	lower lobes	1036 (1016–1080)	1093 (1059–1120)	**0.025**
	ratio UL/LL	0.99 (0.95–1.00)	1.00 (0.99–1.01)	**0.025**
Delta T1 (ms)				
	whole lung	138.0 (94.0–161.0)	149.0 (148.0–181.0)	0.102
	upper lobes	125.0 (104.0–151.0)	154.0 (143.5–173.3)	**0.013**
	lower lobes	154.0 (96.0–171.0)	151.5 (142.5–179.3)	0.255
	ratio UL/LL	0.94 (0.70–1.15)	1.02 (0.89–1.08)	0.689
QCD of T1 room air (ms)				
	whole lung	0.044 (0.036–0.059)	0.029 (0.025–0.036)	**0.002**
	upper lobes	0.041 (0.032–0.054)	0.029 (0.026–0.034)	**0.007**
	lower lobes	0.044 (0.036–0.058)	0.028 (0.024–0.038)	**0.002**
	ratio UL/LL	0.93 (0.77–1.13)	1.03 (0.88–1.11)	0.442
QCD of T1 oxygen (ms)				
	whole lung	0.041 (0.036–0.047)	0.033 (0.025–0.041)	**0.034**
	upper lobes	0.042 (0.035–0.045)	0.028 (0.023–0.040)	**0.034**
	lower lobes	0.041 (0.034–0.053)	0.037 (0.026–0.043)	0.207
	ratio UL/LL	1.03 (0.82–1.08)	0.89 (0.75–0.93)	0.079
QCD of Delta T1				
	whole lung	0.372 (0.308–0.504)	0.277 (0.202–0.379)	0.132
	upper lobes	0.369 (0.296–0.530)	0.256 (0.187–0.365)	0.091
	lower lobes	0.367 (0.289–0.506)	0.306 (0.222–0.401)	0.117
	ratio UL/LL	0.85 (0.77–1.58)	0.90 (0.84–0.96)	0.976
**Regional Fractional Lung Ventilation MRI **				
Median FV				
	whole lung	0.132 (0.105–0.191)	0.181 (0.168–0.233)	**0.027**
	upper lobes	0.118 (0.074–0.181)	0.178 (0.157–0.231)	**0.014**
	lower lobes	0.173 (0.127–0.184)	0.193 (0.170–0.226)	0.204
	ratio UL/LL	0.77 (0.43–1.14)	0.97 (0.86–1.05)	**0.050**
QCD of FV				
	whole lung	0.476 (0.360–0.631)	0.320 (0.289–0.355)	**0.002**
	upper lobes	0.507 (0.381–0.666)	0.317 (0.292–0.352)	**0.0004**
	lower lobes	0.398 (0.306–0.444)	0.307 (0.257–0.372)	0.078
	ratio UL/LL	1.26 (1.15–1.54)	1.02 (0.86–1.20)	**0.024**

Unless otherwise indicated data are median with 25th and 75th percentiles in parenthesis; UL—upper lobes, LL—lower lobes, QCD–quartile coefficient of dispersion. FV–fractional ventilation. *P* values calculated by a 2-sided Wilcoxon test.

**Fig 3:**
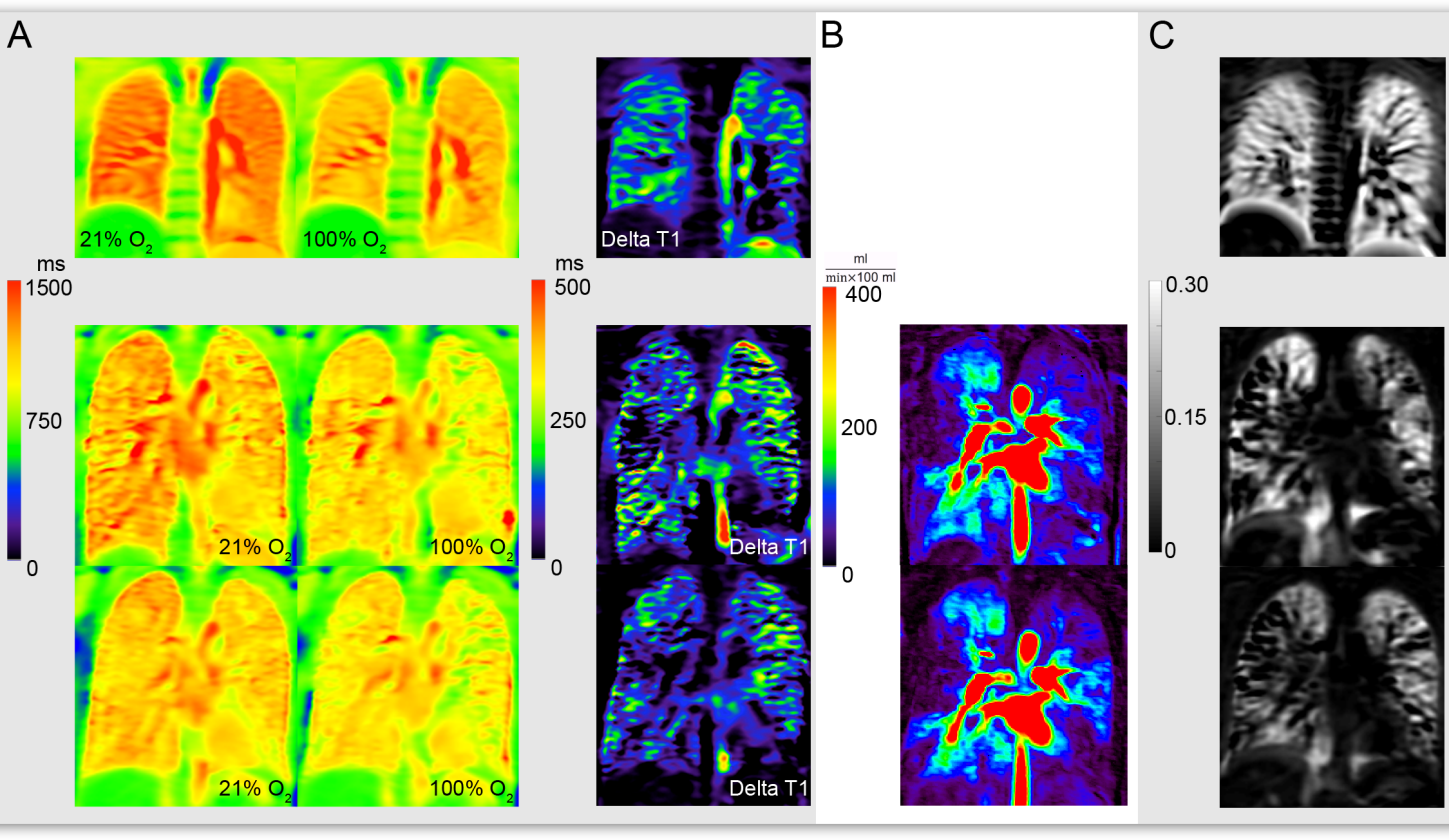
Comparison of a healthy volunteer and a CF patient. First row: healthy volunteer, second row: pre treatment, third row: post treatment; A: room air T1 map, oxygen enhanced T1 map and delta T1 map; B: Calculated perfusion map by using a pixel-by-pixel deconvolution analysis; C: Fractional ventilation map. Regarding the healthy volunteer values are homogeneously distributed throughout the whole lungs. In comparison to that in the CF patient values are lower and more heterogeneous distributed. Hypoventilated and hypoperfused areas can be seen in all three methods, most prominent in the right upper lobe. After treatment an improvement of ventilation and perfusion is not observed.

Median T1-values in the CF group were significantly lower at room air as well as at oxygen on a global as well as on a lobar level. The resulting Delta T1-values were significantly lower for the upper lobes in the CF-cohort [125 (104–151) ms] compared to those in healthy volunteers [154 (144–173) ms] (p = 0.013). The heterogeneity of T1-values at room air was significantly higher in CF patients on a global as well as on a lobar level (p<0.01 for all). At 100% oxygen T1-values were distributed more heterogeneously in the upper lobes. Fig 3 shows a representative healthy volunteer and CF patient of the treatment group.

Fractional ventilation showed significantly decreased median values and higher heterogeneity in the CF-group in the upper lobes and in the whole lung. Additionally the ratio of values of the upper lobes to lower lobes was significantly altered in the CF group for both median values as well as the QCD.

**Fig 4:**
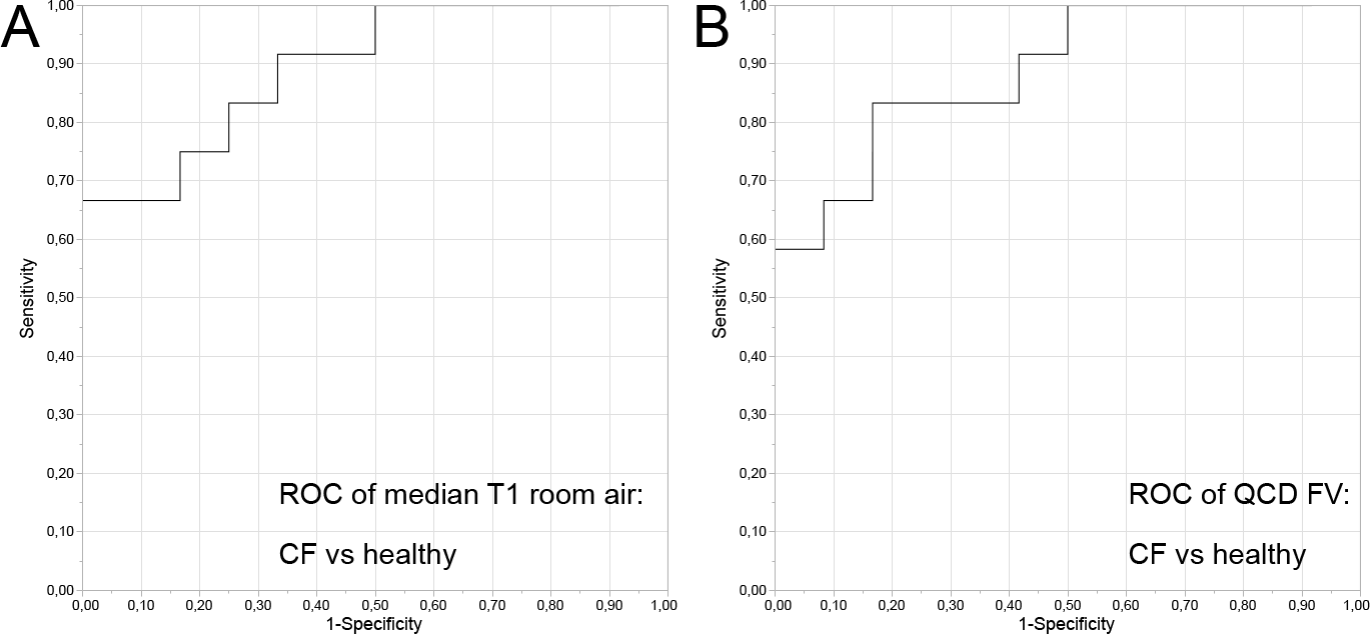
Receiver operator characteristic analysis (ROC). Receiver operator characteristic (ROC) analysis with clinical diagnosis as the criterion showed the ability of functional MRI to discriminate young adults with CF from healthy volunteers. A: Room air T1 (cut point: 1200 ms; area under the curve (AUC) 0.89); B: Quartile coefficient of dispersion of fractional ventilation (cut point 0.351; AUC 0.89).

A receiver operator characteristic (ROC) analysis with clinical diagnosis as the criterion showed the ability of functional MRI to discriminate young adults with CF from healthy volunteers. The highest area under the ROC-curve were calculated for the median T1-values at room air (area under the curve (AUC) of 0.89), and the quartile coefficient of dispersion of fractional ventilation (AUC of 0.89), (Table 3, Fig 4).

**Table 3:** Receiver operating characteristics analysis.

Variable		Cut point	Specificity	Sensitivity	AUC
**Oxygen enhanced functional Lung MRI **					
	Median T1 room air (ms)	1200	1.00	0.64	0.89
	Median T1 room oxygen (ms)	1053	0.92	0.73	0.87
	Delta T1 (ms)	138	0.92	0.55	0.71
	QCD of Delta T1	0.481	1.00	0.36	0.69
	QCD of T1 room air	0.038	0.92	0.73	0.88
	QCD of T1 oxygen	0.039	0.75	0.73	0.77
**Regional Fractional Lung Ventilation MRI **					
	Median FV	0.169	0.75	0.81	0.82
	QCD of FV	0.351	0.75	0.91	0.89

Cut points and area under the curve (AUC) were determined for balanced sensitivity and specificity. FV—fractional ventilation; QCD—quartile coefficient of dispersion.

### Correlation analysis

Correlation analysis showed conclusive results between the assessments of regional perfusion and ventilation using functional lung MRI: Median PBF and Median FV for the upper lobes (r = 0.72, p = 0.01) and QCD of PBF with QCD of FV (r = 0.69, p = 0.02).

Correlations of pulmonary function tests with functional lung MRI parameters or CF-MRI score were not present.

### Functional lung MRI for monitoring hypertonic saline treatment in CF

**Fig 5:**
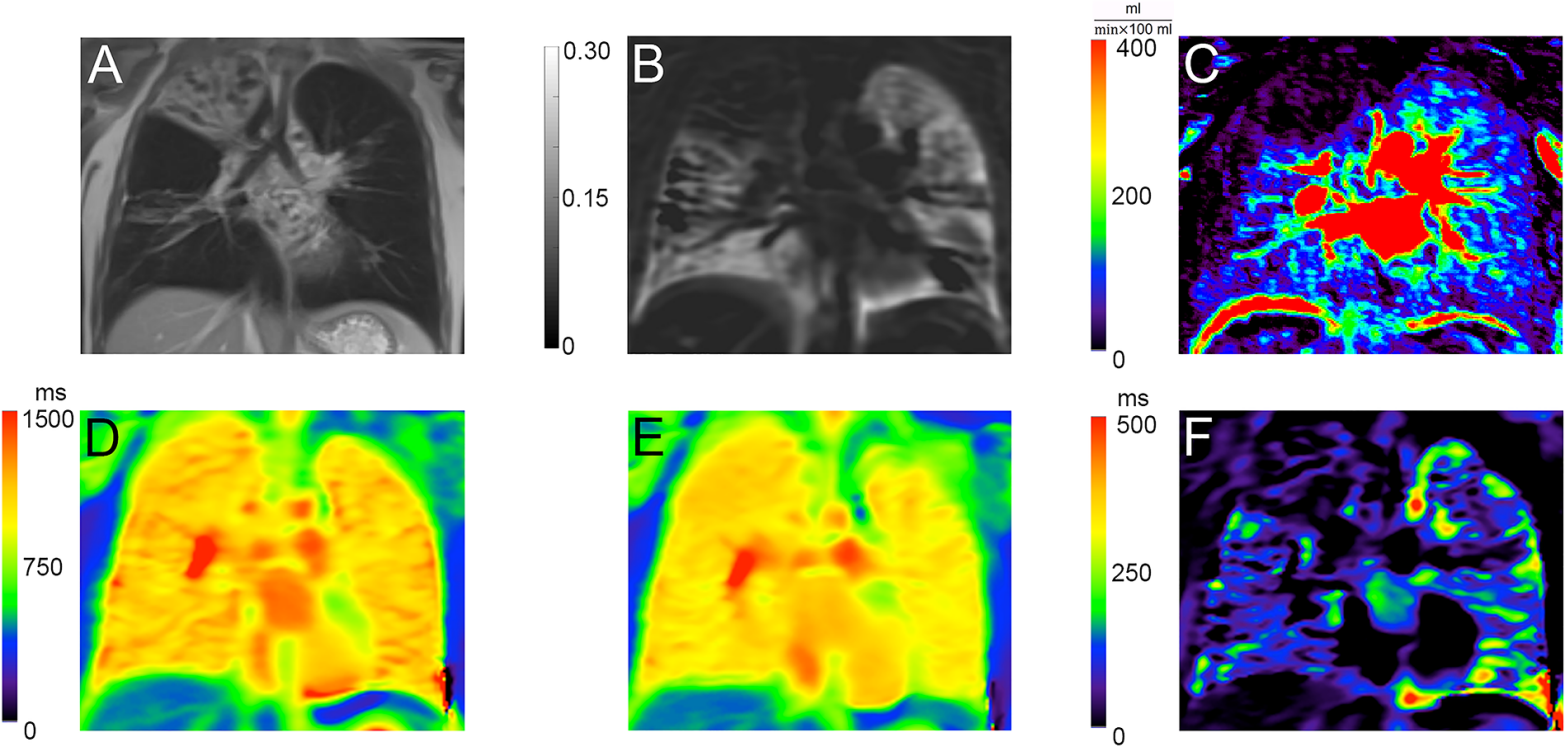
Incidentally discovered consolidation in the right upper lobe in a CF patient. A: T2-weighted; B: Fractional ventilation map; C: Calculated perfusion map; D: Room air T1 map; E: Oxygen enhanced T1 map; F: Delta T1 map.

Results of the MRI-scans of CF patients in the hypertonic saline and control CF group are shown in Table 4: Two hours after a single treatment of hypertonic saline, no significant change of regional lung perfusion or regional ventilation was found in the saline or in the control CF group. However, T1-relaxation was significantly shortened in the saline group at room air (pre 1155 (1098–1202) ms; post treatment CF group 1103 (1062–1161) ms, p = 0.01) and in the control CF group (1^st^ scan 1214 (1084–1231) ms; 2^nd^ scan 1090 (949–1216) ms; p = 0.03) for the whole lung as well as at 100% oxygen for the whole lung. These changes could also be seen on a lobar level. Exemplary ventilation and perfusion maps of a CF patient are shown in Fig 5.

**Table 4:** Functional lung MRI of the CF patient cohort pre and post hypertonic saline treatment vs CF controls.

		HTS treatment (n = 10)			Control (n = 6)		
Variable		Pre	Post	*P* value	1^st^ scan	2^nd^ scan	*P* value
**Cardiac output and pulmonary blood flow (PBF)**							
Cardiac Output (l/min)			5.9 (5.0–6.7)	6.0 (5.1–7.1)	0.49	5.6 (5.2–6.6)	5.2 (4.9–6.3)	0.09
Median PBF (ml/100 ml/min)*							
	whole lung	77.3 (53.5–97.6)	67.7 (55.3–101.9)	0.49	88.2 (76.9–111.8)	79.6 (53.6–108.2)	0.22
	upper lobes	56.4 (43.0–92.5)	55.0 (44.9–98.6)		81.7 (53.9–101.4)	67.1 (44.0–101.2)	0.44
	lower lobes	99.3 (58.1–120.7)	86.9 (63.8–123.8)		107.0 (86.4–130.7)	94.5 (77.4–116.7)	0.22
	ratio (upper/lower)	0.70 (0.54–0.87)	0.69 (0.56–0.92)	0.38	0.70 (0.52–0.98)	0.78 (0.57–0.96)	0.56
Median PBF/cardiac output (ml/100 ml/min/l/min)*							
	whole lung	12.60 (9.39–18.32)	11.08 (8.39–19.23)	0.63	15.61 (12.54–21.62)	15.25 (8.81–19.71)	0.84
	upper lobes	9.95 (7.16–17.21)	9.19 (6.68–18.61)	1.00	14.87 (8.79–19.03)	12.82 (8.30–18.34)	1.00
	lower lobes	15.37 (10.89–20.72)	12.83 (9.92–22.72)	0.70	18.06 (13.57–25.34)	17.25 (11.05–22.69)	0.84
**Oxygen enhanced functional Lung MRI**							
Median T1 room air (ms)							
	whole lung	1155 (1098–1202)	1103 (1062–1161)	**0.01**	1214 (1084–1231)	1090 (949–1216)	**0.03**
	upper lobes	1112 (1097–1192)	1091 (1054–1129)	**0.01**	1169 (1086–1225)	1087 (938–1174)	0.06
	lower lobes	1197 (1103–1211)	1125 (1168–1177)	0.05	1226 (1081–1253)	1091 (958–1223)	0.06
Median T1 oxygen (ms)							
	whole lung	1026 (1004–1064)	993 (978–1023)	**0.02**	1053 (1041–1096)	995 (879–1066)	0.13
	upper lobes	1021 (997–1054)	998 (963–1020)	0.05	1032 (1029–1097)	989 (876–1052)	0.13
	lower lobes	1036 (1015–1077)	1024 (978–1031)	**0.01**	1077 (1050–1094)	1006 (883–1082)	0.13
Delta T1 (ms)							
	whole lung	130.0 (92.5–152.0)	117.0 (87.0–145.5)	0.29	161.0 (99.0–165.5)	125.0 (72.0–150.5)	0.13
	upper lobes	117.0 (95.5–143.0)	114.0 (87.0–121.5)	0.23	125.0 (97.8–160.5)	126.0 (87.5–140.0)	0.31
	lower lobes	135.0 (88.5–165.5)	115.0 (83.0–147.0)	0.57	154.0 (98.0–172.0)	122.0 (50.0–155.5)	0.13
**Regional Fractional Lung Ventilation MRI**							
Median FV							
	whole lung	0.132 (0.099–0.180)	0.129 (0.111–0.190)	0.77	0.142 (0.128–0.166)	0.157 (0.134–0.182)	0.56
	upper lobes	0.118 (0.066–0.164)	0.117 (0.084–0.186)	0.32		0.156 (0.138–0.176)	0.69
	lower lobes	0.177 (0.117–0.196)	0.158 (0.141–0.201)	0.63	0.157 (0.119–0.175)	0.170 (0.132–0.192)	0.44
**CF-MRI-Score**								
Global score (72 pts.)		15 (5–25)	17 (9–26)	0.81	16 (7–30)	16 (3–31)	1.00
Mean scores							
	score of upper lobes (12 pts.)†	3.5 (1.0–5.3)	3.0 (2.3–5.6)	0.53	4.3 (2.1–6.0)	3.3 (0.4–5.8)	0.25
	score of lower lobes (12 pts.)‡	1.5 (1.0–3.5)	2.5 (1.9–3.9)	0.19	2.3 (0.4–5.3)	3.0 (1.1–5.5)	0.50
**Clinical parameters**							
Pulmonary function tests							
	FEV1 (%)	85.0 (63.5–96.5)	72.5 (54.3–90.8)	0.06	79.0 (58.8–91.2)	**-**	-
	LCI	11.5 (10.4–14.3)	12.2 (10.3–14.8)	0.22	11.3 (7.8–16.8)	11.7 (8.4–19.0)	0.22

Unless otherwise indicated data are median with 25th and 75th percentiles in parenthesis

*: values are indicated as ml blood flow / 100 ml lung parenchyma / minute, adjusted for cardiac output: ml blood flow / 100 ml lung parenchyma / minute / l/min cardiac output respectively

†: (Left upper lobe + right upper lobe)/2 (each item 12 pts.)

‡: (Left upper lobe + Right upper lobe)/2.

### MRI score and PFT detect no immediate changes after treatment

Using the MRI-Score, the median global score did not change significantly in both groups. In the treatment group patients were scored with 15 (2–25) points out of 72 points before and 17 (9–26) points after treatment, p = 0.81. The mean difference between the two readouts was -0.22 ± 0.88; the calculated intraclass correlation coefficient was 0.87 for the global score. Pulmonary function tests remained unchanged pre/post in both groups with a trend to a lower FEV1 and higher lung clearance index (LCI) 30 min immediately after inhalation of hypertonic saline (Table 4).

## Discussion

In this study quantitative functional lung MRI parameters were assessed in healthy volunteers and CF patients.

The major results of this study are:

1. Quantitative regional functional lung MRI parameters detect and quantify significant ventilation and perfusion differences between healthy volunteers and young adults with cystic fibrosis.
2. We could show significant correlations between MRI-derived regional perfusion and ventilation measures.
3. Neither MRI nor PFT could detect a significant treatment effect two hours after a single treatment with hypertonic saline. Significant T1 shortening in the lung parenchyma could be observed in both CF groups, which likely reflects residual contrast media in the lung parenchyma applied during the first MRI scan.

Comparing five CF patients to healthy subjects Jakob et al observed shorter T1 times, reduced T1 time differences between different oxygen levels and more inhomogeneous distribution of values. The investigators explained these findings with a reduced oxygen transfer, increased bound-water-fraction (increased collagen in lung tissue) and decreased free-water-fraction (decreased regional pulmonary blood flow) in CF patients [30]. In accordance, in the presented study T1 times in the whole lung at room air as well as at 100% oxygen were significantly shorter and more inhomogeneous in the CF group compared to normal controls, while Delta T1 was lower in the upper lobes only. A possible explanation is that although lung tissue in the whole lung is diseased (shortening of T1 at room air), oxygen transfer is mainly impaired in the upper lobes (small Delta T1). This can be explained by the predominant occurrence of CF-related lung disease, particularly bronchial inflammation and destruction, in the upper lungs [31,32], which is supported by a higher CF score, and impaired and more heterogeneous fractional ventilation measurements in the upper lobes in the presented CF cohort. The underlying mechanisms for this regional variation are still unclear. It has been suggested that the upper lobes are more prone to atelectasis and aspiration pneumonitis [31]. T1 relaxation times and Delta T1 measurements in healthy control subjects are in the range as previously published [23,30,33].

More heterogeneous ventilation reflected by a higher QCD of both oxygen enhanced functional lung MRI and regional fractional lung ventilation MRI in the CF cohort compared to healthy volunteers is in line with ventilation heterogeneity in CF patients observed with hyperpolarized gas MRI[34].

Regional perfusion and ventilation measurements of functional MRI correlate well with each other. This is in line with studies validating Fourier decomposition MR imaging with DCE MRI [22,35], ^3^HE-Imaging [35] and SPECT/CT [36]. However, lung function tests did not correlate with functional lung MRI. This may indicate that quantitative regional lung MRI ventilation and perfusion measures are complementary to FEV1 and LCI measurements and show the potential to add value for CF patient monitoring and management in the future [12,37,38].

Inhalation of HTS has been shown to significantly improve mucociliary clearance in a number of clinical trials [39,40]. Two studies reported improvements in lung function with HTS therapy after 14 days as well as after 48 weeks [8,21]. In the present study the short-term effects of a single treatment with HTS were studied with quantitative functional lung MRI parameters. However, in the presented study neither functional lung MRI nor PFT could detect any treatment response effects in regional or global lung function in young adults with CF 2h after a single treatment with HTS. This is in accordance with Amin et al. who reported recently that 24 h after a single dose inhalation of hypertonic saline PFT could not detect and statistically significant treatment effect using LCI as outcome measure [41].

A significant shortening of T1 values both at room air and 100% oxygen was observed comparing the 1^st^ with the 2^nd^ scan in both CF groups, while heterogeneity of T1 values and oxygenation (Delta T1) did not change significantly. Previously, a high reproducibility of T1 measurements was shown using the same full closed mask system in healthy subjects [23]. Thus, factors affecting the T1 measurement such as the level of inspiration [33,42], ventral-dorsal slice position in the lung parenchyma [23,33,43] and changes of lung perfusion [13] were evaluated: Recently, it was demonstrated that the level of inspiration had only a minimal effect on T1 values[44,45]. Furthermore inflation levels in the CF group trended to be lower on the second MRI scan (which would actually increase T1 values). Slice positioning was comparable using the tracheal bifurcation as an anatomic landmark and the cardiac output measured in the main pulmonary artery as well as parenchymal perfusion assessed by DCE MRI did not differ before and after treatment. Thus, the observed T1 shortening on the second MRI scan performed four (3:41–4:31) hours after the first MRI scan of 3.3(0.3–4.1)% at room air and 1.8(0.1–3.8)% at 100% oxygen (both CF-groups, n = 12) needs to be explained. Recently, a significantly reduced washout of gadolinium-based contrast media in the lung parenchyma was observed using DCE MRI in asthma patients and explained by an increased extravascular space due to interstitial edema or accumulation of fluid within the alveoli and small airways [46]. Also in a reproducibility study (using 0.1 mmol/kg body weight Gd-DTPA) Triphan et al. observed lower T1 values of the lung parenchyma in COPD patients on the second MRI after 23 hours (1124 ms vs 1086 ms; 3,4%)[47]. This finding strongly suggests that the observed T1 shortening in the CF patients in the presented study may be due to residual contrast media possibly due to inflammatory changes in the lung parenchyma of the CF patients. However, gadolinium contrast agent washout in humans depends on many variables (i.e. kidney and liver function, contrast agent group)[48]. The influence of residual contrast media should be taken into account in the design of future studies with multiple time points that use DCE MRI and other functional MRI techniques based on T1 measurements.

A limitation of this study was its relatively small study group of adolescents suffering from CF and therefore the ROC curve analysis has to be interpreted with caution. This distinct group was chosen, because it shows the largest annual loss of FEV1 [49] and therefore would benefit most from improved monitoring with new MRI derived biomarkers. Only three coronal slices were acquired for T1 oxygen enhanced functional Lung MRI and regional fractional lung ventilation MRI covering 4.5 cm of the thorax (approximately 1/3 of the thorax of the CF patients in our cohort). This might have biased the correlation analysis with pulmonary function testing. A possible influence of cardiac movement and pulsation on T1 mapping MRI cannot be excluded. Nevertheless we used an established and reproducible method as described by Jakob et al. without the use of ECG triggering[13,23]. Four CF patients (20%) did not complete the examination due to claustrophobia. This underlines the need of good patient preparation by pediatricians, radiologists and parents in order to foster the compliance in this young patient population.

MRI lung imaging, multiple breath nitrogen washout and spirometry were performed before and two hours after a single treatment with inhaled hypertonic saline. In accordance to Elkins et al. we observed a worsening of PFT parameters immediately after HTS inhalation and functional lung MRI could not show a treatment effect[8]. Thus the chosen time point of the follow-up assessment was likely too early in the present study. This is further supported by two studies of Amin et al. who could show little to no LCI response to HTS inhalation in the short term[41], but a strong response in the longer term[6]. Furthermore bronchodilators could be given in order to reduce early adverse effects of HTS therapy. In this study bronchodilators were not administered to exclude possible confounders.

In conclusion, this study shows the feasibility of functional lung MRI, as a non-invasive, radiation-free tool for visualization and quantification of potential regional treatment effects in patients with CF. Quantitative regional lung MRI ventilation and perfusion measures may be used complementary to the established testing with clinical global lung function parameters.

## Supporting information

S1 File Trial study protocol. (DOC)

S2 File TREND Checklist. (PDF)

S3 File Supporting information. This xlsx file contains each patient’s/volunteer’s measurement data.
(XLSX)
