# Supplementary material for: Functional lung MRI for regional monitoring of patients with cystic fibrosis
Source: PLoS One. 2017 Dec 7;12(12):e0187483. doi: 10.1371/journal.pone.0187483 (PMC5720731; doi:10.1371/journal.pone.0187483)
Supplement: S1 File — (DOC) [file pone.0187483.s001.doc]

**Study Protocol**

**Functional Lung MRI for treatment monitoring in patients with cystic fibrosis**

**Prof. Dr. Vogel-Claussen**: Creation of the functional lung MRI protocol, overall responsible for the study.

**Prof. Dr. Tümmler**: Patient inclusion, hypertonic saline treatment. Pulmonary function measurement and mucociliary clearance measurement responsibility for the study and study design.

**Dr Kaireit, Dr. Marcel Gutberlet, Dr. Renne and Christian Schönfeld**: Development and evaluation of the MRT based ventilation and perfusion maps. Evaluation of MRI based flow measurements. Evaluation of the MRI-based regional Oxygen Transfer Function (OTF) of the lung. Statistics.

**Aim and clinical need:**

Cystic fibrosis (CF) is a complex genetic disease with protean manifestations, the most important being increased risk of chronic lung disease resulting in terminal respiratory failure . CF is an autosomal recessive disorder caused by mutations in the CF transmembrane conductance regulator (CFTR) chloride channel. Defects in CFTR protein function not only impact upon cAMP-dependent chloride secretion but also result in increased epithelial sodium channel- (ENaC-) mediated ion absorption in the superficial airway epithelium . As a consequence, increased water reabsorption across airway epithelial cells leads to extreme dehydration of the airway surface liquid layer, chronic mucostasis, and airflow obstruction . This thickened mucus provides an ideal environment for bacterial infection in the respiratory tract.

Dehydration of the airway surface liquid layer has been implicated as the primary initiating event in CF-related lung disease and therapeutic interventions to improve mucus clearance is a cornerstone of treatment in CF . Such interventions include regular chest physiotherapy, mucolytics, and also aerosolized hypertonic saline (HTS; 3% to 7% NaCl) . HTS is defined as a solution possessing an osmotic pressure greater than that of physiologic isotonic salt solution (0.9% NaCl). Inhalation of HTS has been proposed to significantly improve mucociliary clearance and the popularity of its use has increased on the basis of a number of clinical trials . Several mechanisms have been proposed for the observed effectiveness including changes in the rheological characteristics of the airway mucus, increasing airway surface liquid hydration, inhibition of ENaC, as well as immunomodulatory effects . While a large controlled study reported mild positive effects of HTS on lung function , further studies have hurled it back into the limelight .

Recent advances in magnetic resonance imaging (MRI) technology have enabled functional lung imaging, which has been shown to be feasible in CF patients . To date no clinical imaging test without ionizing radiation has been established to evaluate regional lung function. Therefore the aim of this pilot study is to evaluate if functional lung MRI parameters can add value in comparison with conventional lung function parameters (e.g. FEV1) for monitoring treatment response in young adults (12-20y) with CF undergoing hypertonic saline inhalation treatment.

**Patientent Cohort**

20 young adults with CF between 12 and 24 years of age will be included in the study. This group of adolescences was chosen, because they show the largest annual loss of FEV1. They will be recruited from the outpatient CF clinic. Patients must be in a clinically stable condition with a confirmed diagnosis of cystic fibrosis. Pregnant or breast-feeding women, cigarette smokers or patients with hypertonic saline treatment (HST) within the last 7 days will be excluded. Other exclusion criteria include contraindications to MRI or contrast media (allergy to MRI or CT contrast media, GFR< 30 mL/min/1.73 m^2^) or no (patient or parental) consent to participate. The forced expiratory volume in one second (FEV1), must have a minimum volume of >40 percent predicted for age and gender at the pre MRI lung function test. This study needs to include minors, because they are the target cohort, which would benefit most from improved monitoring with new MRI derived biomarkers to improve survival.

.

**Study type:**

This is an explorative prospective case-control study, where the included CF patients act as control pre HST. To evaluate the effect of an interday variance or a systematic error due to residual contrast agent of the 1^st^ MRI affecting measurements of the 2^nd^ MRI 6 patients will not receive any treatment between the 2 MRI scans.

**Methods:**

All patients receive a pre-treatment MRI scan, lung function test, oxygen saturation measurements during their outpatient day visit. Then they receive a treatment with inhaled hypertonic saline (7%) on the same day according to the SOP 530.00 of the TDV (Therapeutic Diagnositc Nework of the Cystic fibrosis Foundation) to produce induced sputum. Bronchodilators will be not administered, because they could act as confounders. Within 2 hours post treatment, a post-treatment MRI scan will be performed. After this MRI scan the patient will receive another lung function test and oxygen saturation measurements on the same day. Apart from the MRI scan all other tests are part of the routine clinical outpatient visit.

During each MRI scan 0.03 mmol/kg Dotarem will be administered i.v. for lung perfusion imaging. During each MRI scan the patient will receive 100% oxygen at a flow rate of 15 l/min for about 10 min.

**Study design:**

1. CF patient arrives for a day visit in the CF outpatient clinic (1h):

- Lung Function test (Flow-Volume Curve)
- O2 saturation measurements, blood pressure
- Multiple breath washout test

2. CF Patient goes to the MRI suite 1h:

MRI scan pre-treatment:

- Anatomy (HASTE, TIRM and SSFP MRI sequences)
- T1 Mapping and Oxygen enhanced Imaging
- Ventilation and Perfusion weighted Imaging (Fourier decomposition)
- Cardiac function
- Flow measurements in main right and left pulmonary arteries (phase contrast MRI)
- Lung parenchymal perfusion with iv contrast, Dotarem (TWIST MRI).

3. Treatment with inhaled hypertonic saline (7%) (1h)

4. CF Patient goes to the MRI suite 1h:

MRI scan pre-treatment:

- Anatomy (HASTE, TIRM and SSFP MRI sequences)
- T1 Mapping and Oxygen enhanced Imaging
- Ventilation and Perfusion weighted Imaging (Fourier decomposition)
- Cardiac function
- Flow measurements in main right and left pulmonary arteries (phase contrast MRI)
- Lung parenchymal perfusion with iv contrast, Dotarem (TWIST MRI).

5. CF outpatient clinic (1h):

- Lung Function test (Flow-Volume Curve)
- O2 saturation measuremnts, blood pressure
- Multiple breath washout test.

**Parameter:**

Primary outcome variable:

MRI derived pulmonary ventilation and perfusion score

**Secondary outcome variable:**

MRI: T1 value of lung parenchyma, oxygen transfer function, anatomical score (especially mucus plugging). Central flow pulmonary artery flow profile.

Lung clearance index and lung function test parameters.

**Data:**

Data is stored on a password protected research server. The access is only allowed by the research group members.

**Events:**

Very rarely contrast reactions to Dotarem are observed (about 2 in 10 000). The patients will be screened for allergic contrast reactions and excluded from the study to minimize this risk. Also patients not suited to undergo MRI (e.g. Pacemaker, claustrophobia) will be screened for MRI contraindications and excluded from this study. Also patients with known renal failure (GFR <30) will be excluded from this study to minimize the risk of nephrogenic systemic sclerosis.

**Statistics:**

The data will be analyzed using statistical software. Correlation between MRI biomarkers and Lung function parameters will be performed pre and post treatment. Differences of the MRI derived and clinical lung function test will be tested using a two sided Wilcoxon test.

**Changes:**

Changes to the protocol will be submitted for approval to the ethics committee.

**Publication:**

There is no constraint to publish results from this study.

**Literature:**

**1. Wood RE, Boat TF, Doershuk CF. Cystic fibrosis. The American review of respiratory disease. 1976;113(6):833-78. Epub 1976/06/01.**

**2. Boucher RC, Stutts MJ, Knowles MR, Cantley L, Gatzy JT. Na+ transport in cystic fibrosis respiratory epithelia. Abnormal basal rate and response to adenylate cyclase activation. The Journal of clinical investigation. 1986;78(5):1245-52. Epub 1986/11/01.**

**3. Stutts MJ, Canessa CM, Olsen JC, Hamrick M, Cohn JA, Rossier BC, et al. CFTR as a cAMP-dependent regulator of sodium channels. Science. 1995;269(5225):847-50. Epub 1995/08/11.**

**4. Matsui H, Grubb BR, Tarran R, Randell SH, Gatzy JT, Davis CW, et al. Evidence for periciliary liquid layer depletion, not abnormal ion composition, in the pathogenesis of cystic fibrosis airways disease. Cell. 1998;95(7):1005-15. Epub 1999/01/06.**

**5. Boucher RC. Evidence for airway surface dehydration as the initiating event in CF airway disease. Journal of internal medicine. 2007;261(1):5-16. Epub 2007/01/16.**

**6. O'Sullivan BP, Flume P. The clinical approach to lung disease in patients with cystic fibrosis. Seminars in respiratory and critical care medicine. 2009;30(5):505-13. Epub 2009/09/18.**

**7. Wark P, McDonald VM. Nebulised hypertonic saline for cystic fibrosis. Cochrane database of systematic reviews. 2009(2):CD001506. Epub 2009/04/17.**

**8. Amin R, Subbarao P, Jabar A, Balkovec S, Jensen R, Kerrigan S, et al. Hypertonic saline improves the LCI in paediatric patients with CF with normal lung function. Thorax. 2010;65(5):379-83. Epub 2010/05/04.**

**9. Suri R, Metcalfe C, Lees B, Grieve R, Flather M, Normand C, et al. Comparison of hypertonic saline and alternate-day or daily recombinant human deoxyribonuclease in children with cystic fibrosis: a randomised trial. Lancet. 2001;358(9290):1316-21. Epub 2001/10/31.**

**10. Reeves EP, Molloy K, Pohl K, McElvaney NG. Hypertonic saline in treatment of pulmonary disease in cystic fibrosis. TheScientificWorldJournal. 2012;2012:465230. Epub 2012/05/31.**

**11. Elkins MR, Robinson M, Rose BR, Harbour C, Moriarty CP, Marks GB, et al. A controlled trial of long-term inhaled hypertonic saline in patients with cystic fibrosis. The New England journal of medicine. 2006;354(3):229-40. Epub 2006/01/20.**

**12. Donaldson SH, Bennett WD, Zeman KL, Knowles MR, Tarran R, Boucher RC. Mucus clearance and lung function in cystic fibrosis with hypertonic saline. The New England journal of medicine. 2006;354(3):241-50. Epub 2006/01/20.**

**13. Eichinger M, Optazaite DE, Kopp-Schneider A, Hintze C, Biederer J, Niemann A, et al. Morphologic and functional scoring of cystic fibrosis lung disease using MRI. European journal of radiology. 2012;81(6):1321-9. Epub 2011/03/25.**

**14. Puderbach M, Eichinger M, Haeselbarth J, Ley S, Kopp-Schneider A, Tuengerthal S, et al. Assessment of morphological MRI for pulmonary changes in cystic fibrosis (CF) patients: comparison to thin-section CT and chest x-ray. Investigative radiology. 2007;42(10):715-25. Epub 2007/11/07.**
